# Quorum Sensing Activity of *Aeromonas Caviae* Strain YL12, A Bacterium Isolated from Compost

**DOI:** 10.3390/s140407026

**Published:** 2014-04-22

**Authors:** Yan-Lue Lim, Robson Ee, Wai-Fong Yin, Kok-Gan Chan

**Affiliations:** Division of Genetics and Molecular Biology, Institute of Biological Sciences, Faculty of Science, University of Malaya, 50603 Kuala Lumpur, Malaysia; E-Mails: yanluelim@hotmail.my (Y.-L.L.); robsonee@live.com (R.E.); yinwaifong@yahoo.com (W.-F.Y.)

**Keywords:** Cell-to-cell communication, C4-HSL (*N*-butyryl-L-homoserine lactone), C6-HSL (*N*-hexanoyl-L-homoserine lactone), *N*-acyl homoserine lactone (AHL), MALDI-TOF, triple quodruopole liquid chromatography mass spectrometry (LCMS/MS), Pacific Biosciences, RpoD, thin liquid chromatography (TLC)

## Abstract

Quorum sensing is a well-studied cell-to-cell communication method that involves a cell-density dependent regulation of genes expression mediated by signalling molecules. In this study, a bacterium isolated from a plant material compost pile was found to possess quorum sensing activity based on bioassay screening. Isolate YL12 was identified using matrix-assisted laser desorption ionization time-of-flight (MALDI-TOF) mass spectrometry and molecular typing using *rpoD* gene which identified the isolate as *Aeromonas caviae*. High resolution tandem mass spectrometry was subsequently employed to identify the *N*-acyl homoserine lactone profile of *Aeromonas caviae* YL12 and confirmed that this isolate produced two short chain *N*-acyl homoserine lactones, namely C4-HSL and C6, and the production was observed to be cell density-dependent. Using the thin layer chromatography (TLC) bioassay, both AHLs were found to activate *C. violaceum* CV026, whereas only C6-HSL was revealed to induce bioluminescence expression of *E. coli* [pSB401]. The data presented in this study will be the leading steps in understanding the role of quorum sensing in *Aeromonas caviae* strain YL12.

## 1. Introduction

Composting is an aerobic process, which is mainly carried out by a rapid succession of mixed microbial populations, mainly constituted by bacteria and fungi. At different phases of composting, there are vast changes in the microbial diversity involved. The variations in microbial diversity are highly dependent on the types of raw material used in the composting environment as well as the temperature of each of the composting phases [1]. Through the synergistic action of the microorganisms, organic matters are transformed into a humus-like end product. A wide range of bacteria has been found in different compost environments, which includes species of *Pseudomonas*, *Klebsiella*, and *Bacillus* [2,3]. Composting is known to result in various environmental benefits, a number of which are made possible by the actions of the microorganisms within the compost itself, namely bioremediation of contaminated soil and suppression of plant diseases [4–7]. However, the roles of microflora with quorum sensing (QS) activity in composting environment have not been investigated in depth.

QS is an intercellular communication mechanism used by bacteria to control gene expression through the usage of small, diffusible signal molecules termed autoinducers [8]. QS allows behavioral regulation of bacteria according to population density as the transcription of the QS-regulated target genes are triggered only by a threshold level of exogenous autoinducers which indicates presence of a critical cell mass [9,10]. The autoinducers utilized by majority of gram-negative QS bacteria belongs to the *N*-acyl homoserine lactone (AHL) family [11]. Studies conducted have discovered that quorum sensing plays essential role in controlling expression of advantageous bacterial phenotype which complements to the environmental conditions. Several genes have been reported to be regulated by QS, namely conjugal transfers of Ti plasmid in *Agrobacterium tumefaciens*, production of extracellular toxins by *Pseudomonas aeruginosa*, generation of bioluminescence in *Vibrio fischeri and Vibrio harveyi* and production of plant tissue-degrading exoenzymes in *Pectobacterium carotovora* [11,12].

*Aeromonas caviae* is a mesophilic species of the genus *Aeromonas* and it is commonly known as an environmental strain that occur ubiquitously in aquatic environments. In recent years, *A. caviae* has gained a growing clinical recognition as one of the most prevalent causative agent of pediatric gastroenteritis [13,14]. Besides that, an isolated case of urinary tract infection caused by *A. caviae* have also been reported [15]. Besides its role as a clinical strain, *A. caviae* was also discovered to be a potential biocontrol agent against fungal pathogens with its chitinolytic activity [16]. In this study, we report the identification of a QS *A. caviae* strain isolated from compost.

## 2. Experimental Section

### 2.1. Compost Sampling and Processing

A compost sample was collected from a compost pile at Semenyih, Malaysia in 2014. The geographic coordinates of the sampling site is 2°58′18.8″N 101°50′33.5″E. A total of 10 subsamples were collected throughout the compost pile and subsequently inserted into sterile plastic tube. The compost sample was promptly processed upon arrival in the laboratory. Large particulates and coarse organic matters were removed using sterile spatula.

### 2.2. Isolation of Bacteria Strains

Briefly, 1 g of compost sample was mixed with sterile PBS buffer (100 mM, pH 6.5; 10 mL) via vigorous vortex to produce a suspension. The suspension was then serially diluted and subsequently spread onto Luria Bertani (LB) agar and MacConkey agar. Incubation of the agar plates was conducted at 28 °C for 24 h and colonies with observably different morphology was streaked onto new LB agar to obtain pure colonies.

### 2.3. Screening of Bacterial Isolates with Quorum Sensing Activity by Using C. Violaceum as Biosensor

Cross streaking of isolates with C. *violaceum* CV026 on LB agar was performed to screen for QS activity of bacteria isolates [17,18]. Inoculated plates were incubated for 24 h at 28 °C. After 24 h of incubation, development of purple pigmentation on the colonies of *C. violaceum* CV026 suggests that the tested isolate produces short chain exogenous AHLs. *E. carotovora* GS101 and *E. carotovora* PNP22 were used as positive and negative controls, respectively [19,20].

### 2.4. Bacterial Strains and Culture Conditions

The bacterial strains used in this study are as listed in Table 1.

All strains, exclusive of *Escherichia coli* [pSB401] are routinely cultured at 28 °C in Luria Bertani (LB) broth (1% peptone, 0.5% yeast extract, 0.5% NaCl, per 100 mL distilled water) with shaking (220 rpm) or on LB agar at 28 °C. *Escherichia coli* [pSB401] was cultured in LB broth supplemented with tetracycline (50 μL/mL) with shaking. For the purpose of AHL extraction, a modified LB medium buffered with 50 mM 3-[*N*-morpholino]propanesulfonic acid (MOPS) to pH 5.5 [22] was used to culture the bacteria strains.

### 2.5. Bacterial Strain Identification Using MALDI-TOF MS

The identification of bacterial isolate by MALDI-TOF MS [18,19,23] was performed using a Microflex MALDI-TOF (Bruker Daltonik GmbH, Leipzig, Germany) bench-top mass spectrometer equipped with built in software FlexControl software and Bruker MALI Biotyper Real Time Classification software (Version 3.1, Build 65). Freshly cultured bacterial isolates were processed using direct transfer procedure (DTP) in duplicates. Single colony of bacteria were smeared thinly onto a 96-well polished steel target plate followed by addition 1 μL of MALDI matrix (a concentrated solution composed of α-cyano-4-hydroxycinnamic acid in 50% v/v acetonitrile, 47.5% v/v water and 2.5% v/v trifluoroacetic acid) and air-dried. MALDI-TOF MS was performed using the manufacturer's suggested settings. In short, 337 nm nitrogen laser was used to generate ions in a mass range of 2 to 20 kDa. Analysis of the mass spectra was done by comparing to the MALDI Biotyper database. Based on the unique mass spectra generated, identification of microorganisms can be done. Identification results of samples were evaluated according to the spectra value obtained where spectra value between 2.3 and 3 indicates a species-level identification, whereas spectra value between 2 and 2.3 indicates a secure genus-level identification and spectra values between 1.7 and 2 indicates a probable genus-level identification. Spectra values obtained that were lower than 1.7 suggesting an unsuccessful identification. MALDI Biotyper MSP creation method (Bruker Daltonik) was used to assemble the dendrogram [24].

### 2.6. Molecular Typing of Bacterial Strain via RpoD Gene Sequence

Whole genome sequencing of selected isolate was performed using the PacBio RS (Pacific Biosciences; Menlo Park, CA, USA) sequencing platform. Extracted genomic DNA was used to construct a 10-kb SMRTbell library per manufacturer′s instruction and was subsequently sequenced in 4 SMRT cells via Magbead loading. *De novo* assembly of the output sequencing data was performed via the hierarchical genome assembly process (HGap) approach. Furthermore, gene prediction of the assembled genomes was performed using Prodigal v2.60 [25] and the predicted translation product was further compared with NCBI non-redundant protein database to identify RpoD protein sequence. The identified RpoD protein sequence were then used as a query sequence to perform the Basic Local Alignment Search Tool (BLAST) search on the NCBI non-redundant protein sequences (nr) database using the blastp algorithm in order to acquire its homologous sequences. Homologous sequences which have maximum identity score between 97% and 100% were selected as reference sequences. Molecular Evolutionary Genetic Analysis (MEGA) version 5.2 [26] were used to align acquired sequences and also to construct phylogenetic tree from the alignment. The alignment of the query sequence with the acquired homologous sequences were done via the by CLUSTAL_W method whereas construction of the phylogeny trees were performed using the Neighbour-Joining method [27]. The RpoD protein reference sequences of the following strains were obtained from NCBI (accession numbers in parentheses): *Aeromonas caviae* (WP 010675616.1), *Aeromonas hydrophila* 4AK4 (AHE50947.1), *Aeromonas media* (WP 005331904.1), *Aeromonas salmonicida* (WP 021139215.1), *Klebsiella pneumoniae* (WP 023298230.1).

### 2.7. Extraction of AHL from Spent Supernatant

Bacteria cells diluted to turbidity of 0.1 at 600 nm were cultured in LB broth which was buffered to pH 6.5 with 50 mM of MOPS and was incubated in 28 °C with agitation at 220 rpm. The samples were prepared such that cultures in triplicates can be harvested at three time points (8 h, 16 h, 24 h) and be used for extraction. Spent supernatants from each culture of *A. caviae* strain YL 12 were mixed adequately with acidified (0.1% (v/v) glacial acetic acid) ethyl acetate at ratio of 1:1 as described previously [18,28]. The spent supernatants were extracted twice using this method. Following extraction, residual organic solvents were dried in the fume hood. The dried extracts were then reconstituted in 1 mL of acidified ethyl acetate and dried again. Lastly, 200 μL of HPLC grade acetonitrile were added into the dried extracts and vortexed for 3 min before leaving overnight at room temperature. The mixture was then centrifuged at 12,000 rpm for 10 min to remove any insoluble residues.

### 2.8. Bioluminescent Reporter Assay Using E. coli [pSB401] as Biosensor

Infinite M200 luminometer-spectrophotometer (Tecan, Männedorf, Switzerland) was used to perform cell density dependent bioluminescence quantification. Overnight culture of biosensor namely *E. coli* [pSB401] cells was diluted to an OD_600nm_ of 0.1. Then, 250 μL of the diluted *E. coli* [pSB401] cells culture were mixed with 1 μL of extracted AHL and dispensed into a 96-well optical bottom microtitre plates following a previously described method with slight modification [18,29]. Synthetic 3-oxo-C6-HSL (250 pg/μL) was used as the positive control whereas sterile LB broth was used as the negative control in this assay. The bioluminescence assay was conducted for 24 h where bioluminescence and optical density (OD_495nm_) were recorded at every 60 min interval. Results were expressed as Relative Light Units (RLU)/OD_495_ against incubation time.

### 2.9. Characterization of AHL Profile of the Aeromonas Caviae via High Resolution Tandem Liquid Chromatography Quadrupole Mass Spectrometry (LC-MS/MS)

The LC delivery system used was the Agilent 1290 Infinity LC system (Agilent Technologies Inc., Santa Clara, CA, USA) with binary pumps, solvent degasser, thermostated column compartment and diode-array detector, injection volume (2 μL) and the settings used was as described previously [18,19,23]. The column used was an Agilent Zorbax Rapid Resolution High Definition SB-C18 Threaded Column (2.1 mm × 50 mm, 1.8 μm particle size) (Agilent Technologies Inc.). A gradient elution was performed with the mobile phase A being HPLC grade MilliQ water with 0.1% (v/v) formic acid and the mobile phase B being reagent grade acetonitrile added with 0.1 (v/v) formic acid. The gradient profiles used were 80% A and 20% B as the starting mobile phase used for 7 min to proportion of 50% A and 50% B, which continued to 20% A and 20% B at 12 min and progressed to a final proportion of 80% A and 20% B and ran 14 min. The flow rate was set at 0.5 mL/min. Subsequently, Agilent 6490 Triple Quadrupole LC/MS system (Agilent Technologies Inc.) was used to perform high-resolution tandem MS to study the AHL extract [18,20,23]. Electrospray ionization (ESI) was used as the ion source. Precursor ion scanning mode in positive ion mode was used for analysis, where Q1 was set to scan a mass range of *m*/*z* 80 to 400 kDa and Q3 was set to monitor for *m*/*z* 102, the product ion that indicates presence of lactone ring. Analysis of chromatograms and mass spectra were conducted using the Agilent MassHunter software where the extracted ion chromatogram were compared with reference peaks generated from synthetic AHLs standards.

### 2.10. Purification and Elucidation of Biosensor Induction Activity of C4-HSL and C6-HSL Using TLC- Bioassay

Purification of the extracted AHL molecules was conducted using thin-layer chromatography (TLC) as described previously [30]. Dried AHLs were dissolved in 100 μL reagent grade acetonitrile and applied to a reverse phase C_18_ TLC plate (TLC aluminium sheets 20 cm × 20 cm, RP-18 F254s, Merck, Darmstadt, Germany) in a volume of 20 μL per lane. Synthetic AHLs namely *N*-butanoyl-L-homoserine lactone (C4-HSL, 1.0 μg per lane) and *N*- hexanoyl-L-homoserine lactone (C6-HSL, 0.1 μg per lane) (Sigma–Aldrich, St Louis, MO, USA) were used as reference standards whereas the reporter strains used were *C. violaceum* and *Escherichia coli* [pSB401]. Subsequently, chromatography of the TLC plate were conducted using a methanol-water (60:40 [v/v]) solvent system followed by overlaying of LB agar seeded with overnight culture of selected reporter strain upon completion of the chromatography. The overlaid plates were then incubated overnight in a sealed container. Activation of biosensor by individual AHLs is indicated by manifestation of purple spots on *C. violaceum*-seeded LB agar and detection of bioluminescent spots on *E. coli* [pSB401]-seeded LB agar. ChemiDoc™ MP Imaging System (Bio-Rad, Hercules, CA, USA) was used to visualise the bioluminescence expression from TLC/*E. coli* [pSB401] bioassay. The R*_f_* values of the AHL spots obtained were compared with those of the synthetic AHL standards to allow a conjectural identification of the types of AHLs produced.

## 3. Results and Discussion

### 3.1. Sampling and Screening for AHL-Producing Bacteria

Isolation and detection of bacterial isolates with QS was conducted by using compost as the sampling source. The mean temperature of compost was 28 °C during the time of sampling. The compost sample was collected from a compost pile where the raw material constituted mainly of plant materials.

**Figure 1.**
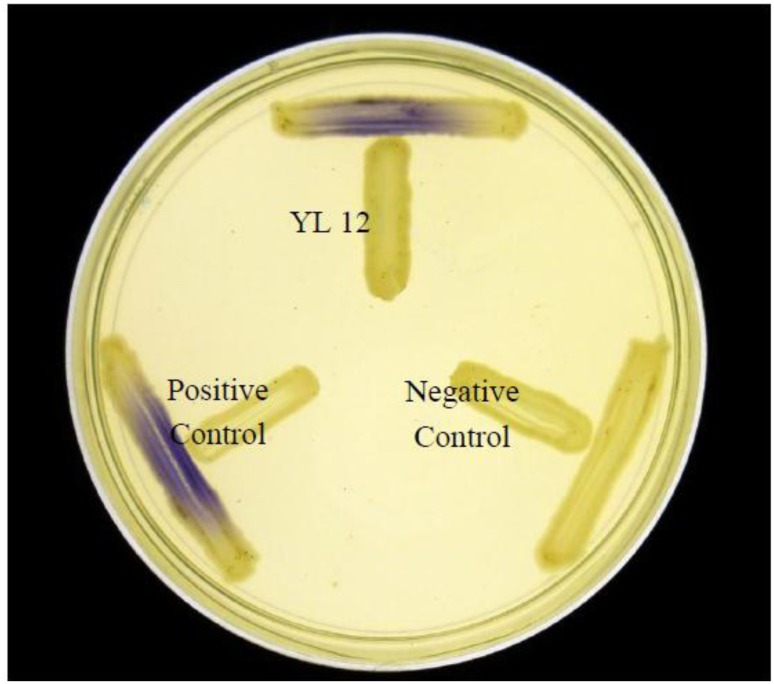
Cross-streaking of strain YL12 with *C. violaceum* CV026 was observed to trigger violacein production in the biosensor. *E. carotovora* GS101 and *E. carotovora* PNP22 were used as positive and negative controls, respectively.

**Figure 2.**
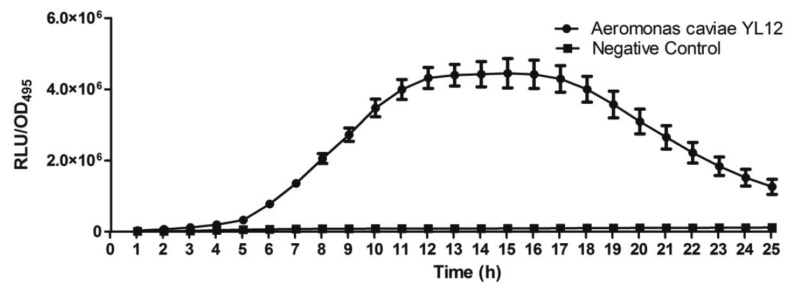
Detection of AHL production by strain YL12 by bioluminescent reporter assay with *E. coli* [pSB401] as biosensor. Graph was plotted as RLU/OD_495_ against time. Increment of RLU/OD_495_ value which was observed to occur exponentially within 13 h confirmed the AHL-producing activity of strain YL12 (circle). Negative control (square) was blank LB broth extract that did not show any detectable activity. Data are presented as means of SEM values from triplicate experiments.

Approximately 80 strains of bacteria were isolated from the compost suspension and all of the strains were preliminarily screened for AHLs production by cross-streaking with *C. violaceum* CV026 as biosensor. *C. violaceum* CV026 is an AHL biosensor that was subjected to mini-Tn*5* transposon mutagenesis which result in a mutation that is defective in production of AHL but retain the capability of producing violacein, a water-insoluble purple pigment, when exogenous short chain AHLs are detected [17]. In the cross-streaking experiment conducted, strain YL12 triggered CV026 violacein production (Figure 1), which suggests that YL12 produces diffusible short chain AHLs. In order to confirm QS activity of YL12, further QS analysis in the form of bioluminescent reporter assay was conducted and YL12 demonstrated a cell density-dependent induction of bioluminescence (Figure 2).

### 3.2. Strain Identification of Strain YL12 using Matrix-Assisted Desorption Ionization Time-of-Flight (MALDI-TOF)

MALDI-TOF mass spectrometry is now routinely used for bacterial identification [23]. The approach to taxonomic characterization of bacteria using MALDI-TOF-MS is by comparing the ions observed in the resultant spectrum to the MALDI Biotyper database. Dendrogram generated from MALDI-TOF MS based on spectral analysis of strain YL12 along with other *Aeromonas* spp. within the Bruker database was used to visualise the taxonomic classification of strain YL12. Strain YL12 was observed to match closely to *A. caviae* 60 PIM. The different colours of the branches represent distinct clusters among the microorganisms in the database.

**Figure 3.**
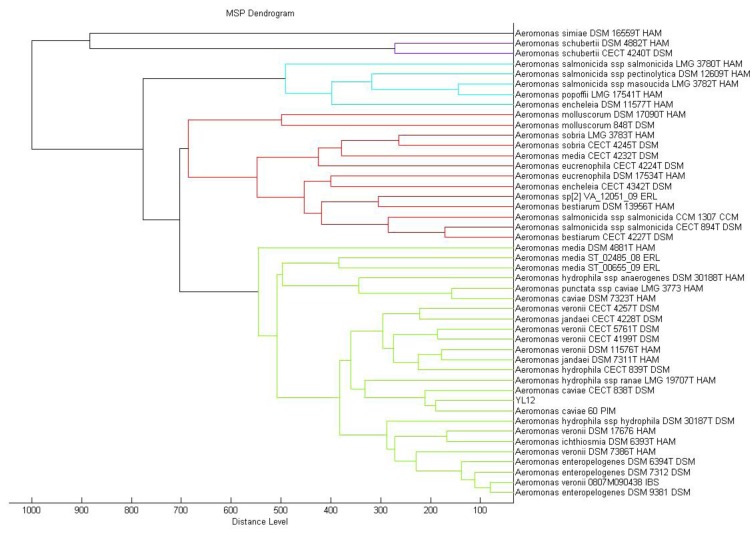
MALDI-TOF MS identification of strain YL12. The different colours of the branches represent distinct clusters among the microorganisms in the database.

In this study, the result generated from the MALDI-TOF-Bioyper Real Time Classification software showing the closest match to the M12 isolate is *Aeromonas caviae* with a best match score value of 2.430, while the second best match at a score value of 2.378 was *Aeromonas hydrophila*. A dendrogram created using the MALDI-Biotyper MSP software also showed that M12 isolates clustered closely to *A. caviae* strain 60 PIM (Figure 3). MALDI-TOF MS used with a reliable database was demonstrated to be a reliable method for the identification of aeromonads [31].

### 3.3. Molecular Identification of A. caviae YL12 via RpoD Gene Sequence

**Figure 4.**
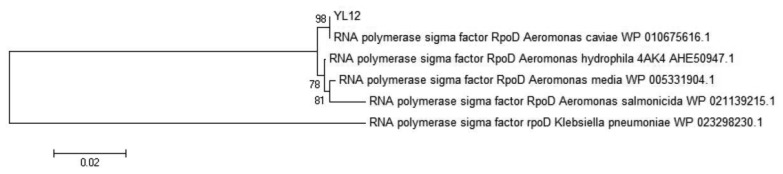
The Phylogenetic Analysis of Strain YL12.

Identification of *Aeromonas* species had been reported to be a challenging task where several studies have highlighted the limitation of both phenotypic identification method as well as commercial identification systems in identification of aeromonads till the species level, particularly in the environmental isolates [32–35]. In view of this problem, molecular approaches had become the preferred tool to perform species identification of the aeromonads. RpoD gene, the single-copy housekeeping gene which encodes the sigma70 factor, was demonstrated in several literatures to be a better molecular marker to be used in the study of phylogenetic and taxonomic relationships [35–37]. The deduced RpoD protein sequence (619 amino acids) was used as query sequence to build a phylogenetic tree. Based on the phylogenetic analysis, the closest relative to YL12 was *A. caviae* with very high bootstrap value (Figure 4). RpoD protein sequence of *Klebsiella pneumoniae* was used as an outgroup. The optimal tree with the sum of branch length = 0.20177266 was constructed. The percentage of replicate trees in which the associated taxa clustered together in the bootstrap test (out of 1,000 replicates) was shown next to the branches [38]. The evolutionary distances were computed using the Poisson correction method [39] and are in the units of the number of amino acid substitutions per site. The analysis involved 6 amino acid sequences. All positions containing gaps and missing data were eliminated. There were a total of 611 positions in the final dataset. Evolutionary analyses were conducted in MEGA5.2 [40]. The evolutionary history was inferred using the Neighbor-Joining method [27].

### 3.4. AHLs Identification by LC-MS/MS

**Figure 5.**
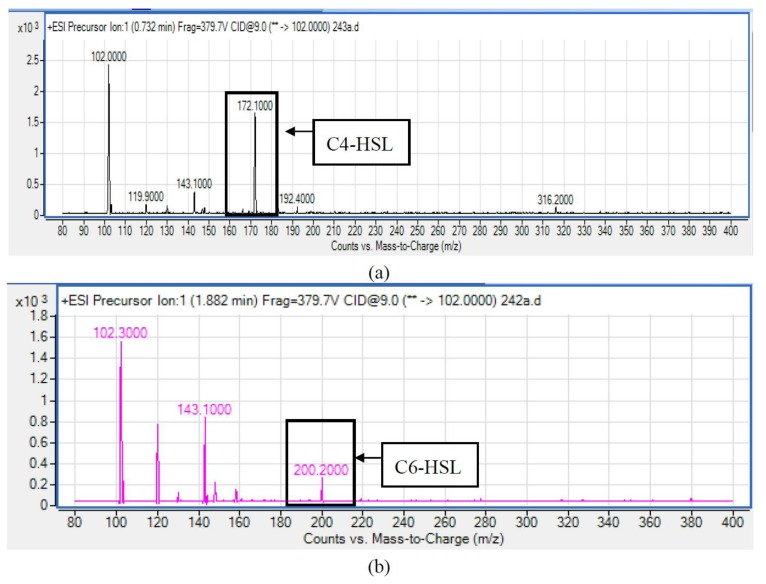
Mass spectrometry analysis of extracted AHL from spent supernatant of YL12 harvested at the 24th hour incubation. (**a**) C4-HSL (*m*/*z* 172.100; retention time: 0.732 min; abundance: 1643.48) and (**b**) C6-HSL (*m*/*z* 200.200; rentention time: 1.882 min; abundance: 261.54).

**Figure 6.**
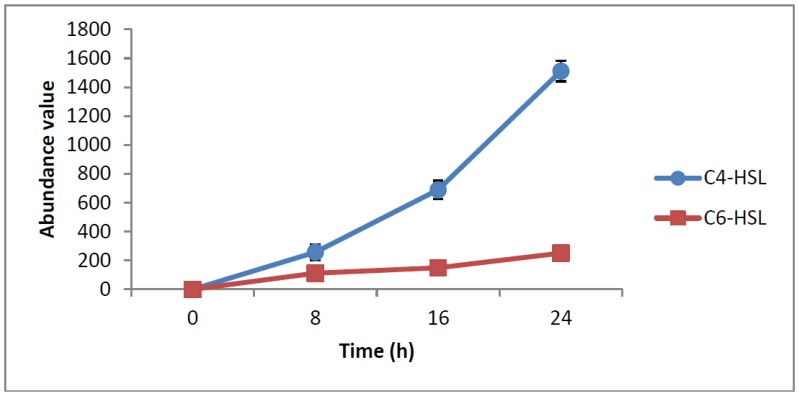
Abundance value of C4-HSL (●) and C6-HSL (▪) collected from culture harvested at three separate time points (8 h, 16 h, 24 h;. C4-HSL were found to increase exponentially from 8 h to 24 h whereas C6-HSL shows a relatively slight increment during the same incubation period. Data are presented as means of SEM values from triplicate experiments.

HPLC fractionation followed by MS analysis of the spent culture supernatants extracts of strain YL12 collected were performed to identify the types of AHLs secreted as well as to determine the effect of increased cell density on the accumulated amount of each AHLs produced. All samples were detected to contain two fractions with retention times corresponding to the synthetic C4-HSL (*N*-butyryl-L-homoserine lactone) and C6-HSL (*N*-hexanoyl-L-homoserine lactone) (Figure 5). In addition, analysis of the collisionally induced spectra of all the identified fractions demonstrated the presence of the major product ion (*m*/*z* value 102) of most AHLs, this detection further affirmed that the fractions detected are AHL molecules (Figure 5). Further, analysis of the abundance value of C4-HSL and C6-HSL detected in each sample showed that the abundance of both AHLs are relatively dependent to the length of incubation period of the respective culture where a longer incubation period coincides with increased abundance of both AHLs. Interestingly, abundance of C4-HSL was shown to increase exponentially from 8 h to 24 h whereas the abundance value of C6-HSL only increased slightly over the same period of time (Figure 6). This finding is in agreement with previously reported studies which demonstrated that C4-HSL is the principal AHL produced in aeromonads, where both LuxI homologue quorum sensing proteins of this species, AhyI and AsaI, were found to produce C4-HSL while C6-HSL is produced in a relatively lower level [30,41]. The pattern of elevation in amount of C4-HSL correlates with its reported role in regulation of cell-density dependent expression of pathogenesis-related phenotypes in this species such as biofilm production, serine protease and metalloprotease activities [42–44]. Regulation of the coordinated expression of these virulence factors *via* QS system is imperative to enhance the pathogenicity of this bacterium by allowing the bacterium to evade host's defense response while mounting effective attacks. Despite the extensive investigation of QS regulated expression of virulence factors in clinical isolates, the role of QS in aeromonads from environmental isolates, particularly from compost, are scarce. Understanding of AHL profile of *A. caviae* from compost isolates can lead to further inspection of the role of QS in the biology of this bacteria and its interaction with its niche.

### 3.5. Characterization of Biosensor Induction Activity of C4-HSL and C6-HSL Using TLC Bioassay

**Figure 7.**
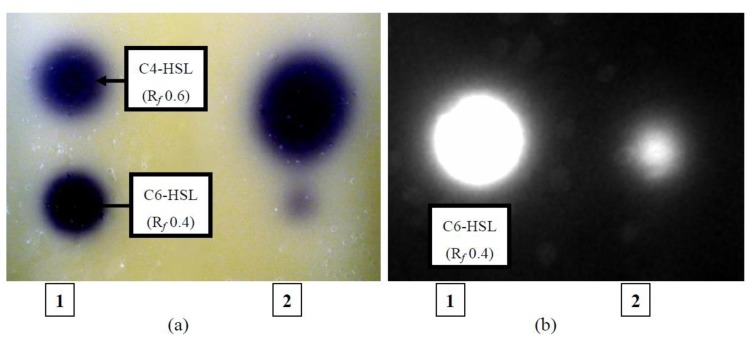
TLC bioassay of spent supernatants extracts of YL12. (**a**) TLC/CV026 bioassay shows that YL12 extracts (lane 2) were chromatographed into two distinct spots with R*_f_* value of 0.4 and 0.6, which corresponds to the R*_f_* values of the synthetic standards (lane 1) used, namely C4-HSL (R*_f_* value of 0.6) and C6-HSL(R*_f_* value of 0.4). (**b**) TLC/ *E. coli* [pSB401] bioassay showed only one bioluminescent spot in both lane 2 (YL12 extracts) and lane 1 (synthetic standard) with R*_f_* value of 0.4 following incubation at 37 °C.

To determine whether AHLs extracted could induce activation of biosensors, bioassay of the AHL extracts were done using *C. violaceum* CV026 and *E. coli* [pSB401] used in conjunction with TLC (Figure 7).

The result obtained revealed that both C4-HSL and C6-HSL produced by YL12 were able to activate CV026, a biosensor which responds to presence of short chain AHLs rapidly (with four to eight side chains) with production of purple violacein pigments [17]. The assay also further confirmed the results obtained from LC-MS/MS where C6-HSL is produced in relatively lower abundance compared to C4-HSL as visualized by the vast difference in the size of the spots produced. On the contrary, the *E. coli* [pSB401]/TLC bioassay shows that only C6-HSL in the solvent extracts of YL12 were detected to be able to activate bioluminescence expression of this biosensor. This finding is in agreement with the previously reported characterization of *E. coli* [pSB401], a biosensor developed from *luxCDABE* operon of *Photorhabdus luminescences*, where the biosensor express good sensitivity towards C6-HSL, even at low concentrations, and have minimal efficacies towards C4-HSL as well as AHLs with long side chains (C10- and longer) [45].

## 4. Conclusions

We report here the identification of a compost isolate, *A. caviae* strain YL12 which produced two short chain AHLs, namely C4-HSL and C6-HSL, and the production of each AHL was discovered to be cell-density dependent. This leads to the hypothesis that the QS system in *A. caviae* YL12 is likely to mirror the QS systems of other *Aeromonas* sp., hence suggesting possible similarities in QS-regulated gene expression. Further research is underway to identify the gene responsible for the QS activity of *A. caviae* YL12 as well as study the physiological activity regulated by the signalling molecules on this bacterium.

## Figures and Tables

**Table 1.** Strains used in this study.

**Strain**	**Description**	**Source/Reference**
*Chromobacterium violaceum* CV026	Double mini-Tn*5* mutant derived from *C. violaceum* ATCC31532 serves as a biosensor which formation of purple violacein on its colonies indicate presence of short chain exogenous AHL molecules.	[17]
*Erwinia carotovora* GS101	Served as positive control in AHL production screening test due to its capability of producing AHL molecules that is detectable by C. *violaceum* CV026	Gift from Prof. Paul Williams
*Erwinia carotovora* PNP22	Served as negative control in AHL production screening test as it lacks AHL molecules production.	Gift from Prof. Paul Williams
*Escherichia coli* [pSB401]	A pACYC184-derived mutant developed from *Photobacterium fischeri* [ATCC 7744] which functions as a AHL biosensor as it produce bioluminescence in the presence of short chain AHL.	[21]
*Aeromonas caviae* strain YL 12	Compost isolates	This study
